# Coping Methods and Satisfaction with Working from Home in Academic Settings during the COVID-19 Pandemic

**DOI:** 10.3390/ijerph191912669

**Published:** 2022-10-03

**Authors:** Jimmy Stephen Munobwa, Fereshteh Ahmadi, Saeid Zandi, Natalie Davidsson, Sharareh Akhavan

**Affiliations:** 1 Department of Social Work and Criminology, Faculty of Health and Occupational Studies, University of Gävle, 80176 Gävle, Sweden; 2 Faculty of Literacy, Rowan-Cabarrus Community College, Salisbury, NC 28146, USA

**Keywords:** coronavirus, enforced telework, higher education, home confinement, job satisfaction, coping strategies, workplace health, occupational health, telecommuting, remote work

## Abstract

In this paper, we examined how university staff and students coped with challenges related to working or studying from home during the COVID-19 pandemic, and the level of satisfaction with working from home. An online survey was conducted among faculty, staff, and students at universities in 24 countries (*n =* 674). The results show that over 80% of the respondents used multiple coping methods. Three clusters of coping methods were generated through factor analysis: (1) social and health factor, with focus on personal health and the social surrounding, (2) activity factor, i.e., being busy with work or studies, finding up-to-date information about COVID-19, while thinking about what one could do rather than what one could not do, and (3) public health factor, which meant trusting health authorities while avoiding misinformation from sources such as social media. Furthermore, 56% of the respondents were very or somewhat satisfied with working from home. Differences in the methods of coping and satisfaction with working from home highlight the need for employers to prepare for working from home beyond the COVID-19 pandemic.

## 1. Introduction

The world was hit by the COVID-19 pandemic in 2020 resulting in subsequent lockdown in most countries. Other preventive strategies were implemented such as mask-wearing, social distancing, and frequent handwashing [1]. With the onset of the lock-down, employers encouraged working from home or other premises other than the usual workplace. University faculty/staff and students were among the groups affected by working from home. Working from home changed the working environment for many employees, presenting both challenges and opportunities for work life [2]. People in academic settings faced a new reality with the closure of campus-based in person activities, and the shift of teaching to different online platforms [3,4]. College and university faculty/staff and students practiced social distancing by working and studying from home.

Earlier research shows benefits of working from home, e.g., increased autonomy and flexibility [5], yet research among academics in the context of the COVID-19 pandemic is limited. Some empirical studies indicate that working from home during the COVID-19 pandemic has increased productivity for some academics, and many are positive about working from home in the future [6]. However, there is evidence from different contexts that associates working from home during the COVID-19 pandemic with negative consequences, e.g., reduced productivity [6], physical ill-health [7] and mental ill-health [3,7,8,9,10]. Therefore, we cannot ignore the negative consequences of the pandemic; a “mental health spill over” can be anticipated now and in the future [11].

Some recommendations were proposed to help the academic population during the COVID-19 pandemic, e.g., creating clear routines for home working, having work life balance, simplifying communication, networking, and using available remote platforms and programs for teaching [12,13,14]. Personal, and technology-related factors, e.g., internet access and family-work conflict mediated the negative effects of working from home [15]. Therefore, academics’ individual efforts seemed to play a big part in how they dealt with the challenges related to working from home during the COVID-19 pandemic.

Coping strategies can alleviate the psychological burden associated with stressful events [16,17]. In the context of COVID-19 and academics, research shows that several methods of coping have been useful. The role of social support during lockdown and working from home is important. Many studies show that academics have utilized contact with family and friends to deal with the stresses of the pandemic and working from home [18,19]. The importance of a support network, e.g., friends, family and work colleagues has also been highlighted in other occupational settings [20,21]. Additionally, meaning-making coping has had an important place in academics’ effort to cope with COVID-19. Using nature as a coping resource and considering life as integrated to a greater whole are significant for coping with COVID-19 among the academics, and in other traumatic situations [2,21]. Additionally, religious coping methods have been used, especially in the context of Muslim academics [22,23]. Moreover, theory and research show that individuals who perceive stressors positively, e.g., as an opportunity for growth, seem better prepared to deal with life challenges [17,24]; this is true in the context of COVID-19 [25,26]. Additional coping strategies generally include having a positive attitude, self-reliance, and self-care [20], although these have not been studied in the context of academics.

Job satisfaction and coping methods used to deal with challenges at work can be understood through models that explain the interaction between job demands, and resources that employees have to meet the demands. One such model is the Demand-Control model by Karasek [27]; this model postulates that working with high demands and low control over job tasks leads to job strain. With more control, employees are often able to cope with the high job demands, thereby avoiding strain. Having control implies employees’ ability to decide how job tasks are conducted and being able to use their skills in task execution [27,28]. Karasek and Theorell refer to this as the decision latitude [27,28]. Several studies demonstrate the applicability of the Demand-Control model [28,29,30].

Although modified to the Job-Demand Control-Support model through the addition of a social support dimension [31,32], the model is insufficient on its own to explain job satisfaction and health outcomes because it assumes centrality of work overload and lack of autonomy as a source of job strain [33]. A modified theoretical approach to understanding the relationship between job demands, satisfaction and employee wellbeing is the Job Demands-Resources Model [33,34]. The model divides job-related characteristics into two, (1) job demands, which many be physical, cognitive, or emotional and (2) job resources, e.g., physical, social, or organizational aspects that alleviate job demands and ensure attainment of work goals [33,34]. Thus, control/autonomy is just one of many aspects that buffer wellbeing and job satisfaction. However, we note that previous research and theoretical perspectives are used only in an indicative manner to provide inspiration in the context of discovery.

Research shows that working from home can increase control over the job, because employees decide how to apportion time between work and family [5]. However, there are contradicting results on how working from home translates into positive health outcomes. For example, Gonzáles Ramos and García-de-Diego [35] found that oscillating between domestic labor and paid work during the lock down led to poor well-being, because it was difficult to concentrate on work. Nevertheless, for women, increased control and work–life balance was positively related to general health much more than men. Therefore, some differences based on gender seem to exist. The “flexibility stigma”, i.e., the desire not to work from home, seemed eradicated by the COVID-19 pandemic, because both female and males were more positive to working from home [24]. In the context of the COVID-19 pandemic, employees in academic setting have enhanced wellbeing and job satisfaction by being proactive and thinking about what they can do about the situation, rather than focusing on what they cannot do [20,26,36]. Therefore, several coping methods must be considered when studying coping with the challenges of COVID-19 pandemic.

Whereas there is research on how academics are generally dealing with the COVID-19 pandemic, there are gaps in research specific to working from home. Moreover, most research is based on local country contexts. There is a paucity of research that addresses working from home and coping at an international level. In this paper, we examined the methods through which university staff and students coped with challenges related to working/studying from home during the COVID-19 pandemic. Additionally, we investigated the level of satisfaction with working from home during the pandemic.

Specifically, we addressed the following research questions:

**Question 1**: What coping methods do academics use to deal with the challenges of working from home during the COVID-19 pandemic?

**Question 2:** To what extent are academics satisfied with working from home during the COVID-19 pandemic?

## 2. Materials and Methods

A quantitative research design method was used for this study. The authors used an online survey to examine university faculty, staff, and students’ methods of coping and job satisfaction with working from home during the COVID-19 pandemic. The study targeted university academics across different countries using convenient sampling (*n* = 674). The countries included Austria, Bangladesh, Denmark, Finland, France, Germany, India, Iran, Italy, Malaysia, Malta, Norway, Philippines, Portugal, Saudi Arabia, Singapore, South Korea, Sweden, Switzerland, The Netherlands, The UK, Tunisia and Turkey. Table 1 demonstrates the characteristics of the respondents.

An online questionnaire was used for data collection. The link to the online survey was e-mailed to faculty members, students and university staff working at different universities across different countries in May 2020. The email and online survey contained an invitation letter explaining the research aim, procedure, and ethical considerations. The data were collected between June and December 2020.

**Figure 1:**
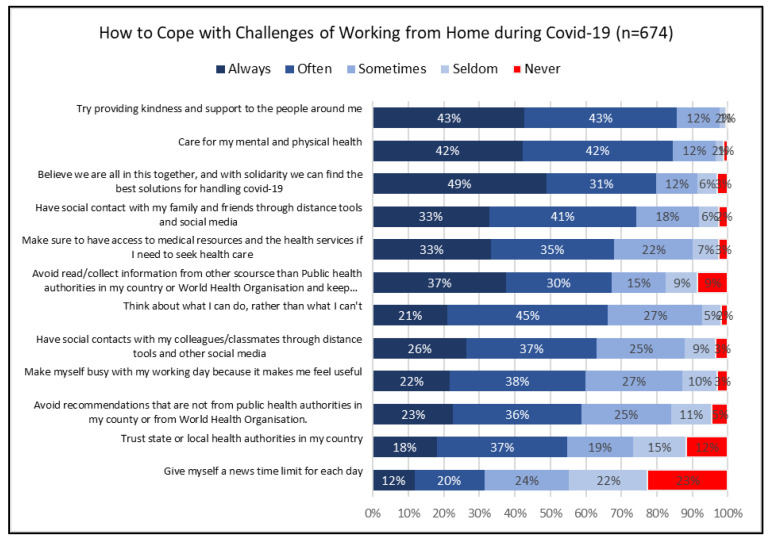
Methods of coping with challenges of working from home.

No pre-existing questionnaire was used for measurement. The COVID-19 pandemic created new challenges and working arrangements for academics and other occupational groups. The challenges created an urgent need to study our research topic; thus, we constructed a questionnaire with items relevant to working from home during the COVID-19 pandemic. In the questionnaire, we asked four questions: (1) if the respondent worked more than contracted (responses *yes* or *no*); (2) how satisfied the respondent was with working from home (responses *very dissatisfied, somewhat dissatisfied, neither satisfied nor dissatisfied, somewhat satisfied,* and *very satisfied*); (3) how the respondent rated his/her general health (responses on a scale *poor, fair, good, very good* and *excellent*); and (4) how the respondent coped with challenges related to working from home (responses on a scale *never, seldom, sometimes, often* and *always* in relation to 12 methods of coping included in the questionnaire, see Figure 1) with a Cronbach’s Alpha value of 0.726 (good level). For this paper, we used responses to question 1, 2 and 4.

We used a single-item measure of job satisfaction to keep the questionnaire simple and short. Nevertheless, a single-item measure of job satisfaction can yield reliable and valid results [35]. The methods of coping included in the questionnaire were adapted from our extensive international research on coping in times of crisis [21,37,38,39,40,41], the revised Brief RCOPE questionnaire [40], and the budding research on COVID-19 that was available at the time. In addition, we borrowed from the Demand-Control model [27,28] and it’s later versions, with focus on possible resources necessary to reduce work stress in the context of working from home.

It was not our intention to study differences in coping and satisfaction with working from home in different countries or socio-cultural settings. Rather, we intended to focus on what the academics had in common as an occupational group. We did not translate the questionnaire based on the assumption that the respondents, being academics, had at least professional working proficiency in the English language.

Based on the sample of 674 respondents, the margin of error for this study is +/− 3.9 percentage units for results around 50%, as well as 2.3 percentage units for results around 10 or 90%. See Table 1 for the sample description.

Statistical analysis was conducted using IBM SPSS Statistics 27. The sample was not weighted to reflect the actual academic population of staff or students of which it was representative. We conducted exploratory factor analysis to identify the underlying coping methods, i.e., factors measured by the coping methods we studied. In this way, we could identify coping methods that belonged together, thereby condensing them into fewer latent variables (factors). Factor analysis identified interdependent coping methods, i.e., if one coping method *x* was normally mentioned together with another coping method *y*, then *x* and *y* were factored together. We used varimax rotation and all coping methods were included in the analysis. Varimax rotation takes the correlation between the coping methods and factors and maximizes the sum of their variance. We used varimax rotation because it can produce simpler and easy-to-interpret factor solutions. The 12 coping methods were originally not formulated to construct an explanatory model; thus, the intention was not to conduct a confirmatory factor analysis

The Declaration of Helsinki was considered when conducting the study. Respondents were informed about voluntary participation, anonymity, and data usage and storage. Ethical approval was granted by the Swedish Ethical Review Authority, for elements of the study linked to data collection, analysis, usage and storage in Sweden (Reg. no. 2020/02368 9).

## 3. Results

The results are presented in four sub-sections—coping with challenges of working from home, time input when working from home, satisfaction with working from home, relationship between coping methods and satisfaction with working from home.

### 3.1. Coping with Challenges of Working from Home

The four mostly used methods of coping included “providing kindness and support to the people around me”, “caring for my mental and physical health”, “believing that we are all in this together, and with solidarity we can find the best solutions for dealing with COVID-19” and “having social contact with my family and friends through distance tools and social media”. More than 80% of the respondents used 9 of the 12 methods of coping studied. The three methods of coping that were least used included “giving myself a news time limit for each day”, “trusting state or local health authorities in my country” and “avoiding recommendations that are not from public health authorities in my county or from World Health Organization (WHO)”. Forty-five percent (45%) of the respondents seldom or never had a daily news time limit. Twenty-seven percent (27%) seldom or never coped through trusting the state or local authorities, while 18% seldom or never avoided reading/collecting information from sources other than national public health authorities or WHO (See Figure 1).

Based on factor analysis, three clusters of methods of coping emerged. The first cluster, labeled *social and health factor,* focused on personal physical and mental health, as well as the surrounding. Methods of coping in this cluster included taking care of one’s physical and mental health, provide kindness and support to people around, making sure one had access to medical care when needed, believing that COVID-19 was a communal problem, showing solidarity, having social contact with work colleagues or classmates, and keeping social contact with family and friends. The second cluster was labeled *activity factor* and focused on being active while dealing with the COVID-19 pandemic. This included giving oneself a news time limit for each day, making oneself busy with the working day to feel useful, thinking about what one could do, rather than what one could not do, and reading/collecting information from public health authorities or WHO and keeping oneself updated with public health news. The third cluster, named *public health trust factor*, included trusting local or state health authorities, or avoiding misinformation in social media and recommendations from sources other than public health authorities in the country” (See Table 2).

### 3.2. How Much Academics Worked during the COVID-19 Pandemic

**Figure 2:**
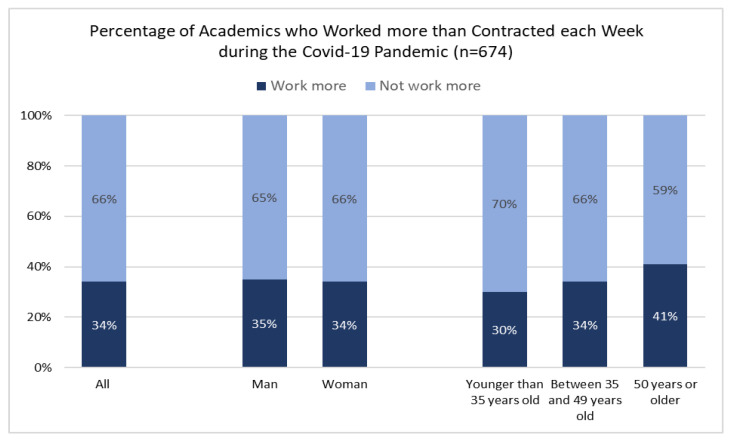
How much academics worked during the COVID-19 pandemic.

The majority of the respondents did not work more than contracted during the COVID-19 pandemic. However, about three in ten (34%) worked more than contracted each week during the COVID-19 pandemic. Respondents aged 50 years and above were overrepresented among those who worked more than contracted (See Figure 2). There were no noteworthy differences based on gender.

### 3.3. Satisfaction with Working from Home

**Figure 3:**
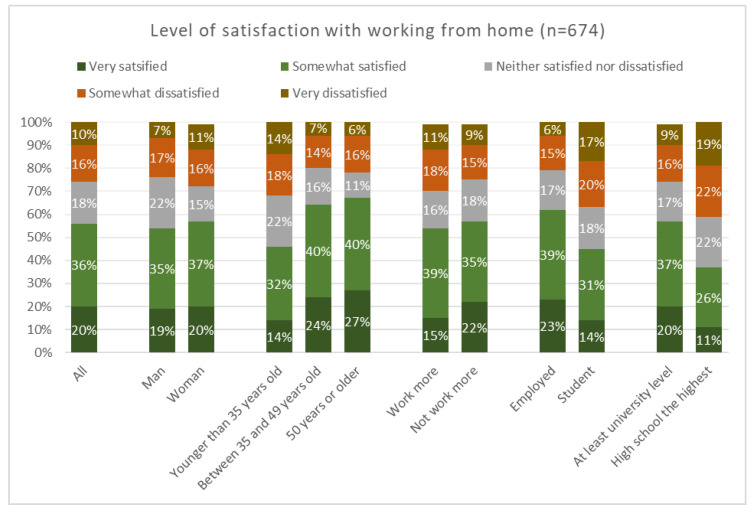
Level of satisfaction with working from home.

Most respondents (56%) were satisfied or somewhat satisfied with working from home. Slightly more women than men were satisfied with working from home, although the observed difference was minimal. Respondents under 35 years of age were much less satisfied with working from home compared to other respondents. Respondents who did not work more than contracted were more satisfied with their work situation compared to those who worked more. University employees were much more satisfied (62%) compared to students (45%). Additionally, respondents with higher education were more satisfied with working from home (57%) as compared to their colleagues with a lower education level (37%) (See Figure 3).

## 4. Discussion

This paper set out to examine the ways in which university staff and students coped with challenges related to working/studying from home during the COVID-19 pandemic. Additionally, the study investigated satisfaction with working from home during the pandemic. The results show that over 80% of the respondents used 9 of the 12 methods of coping studied. The mostly used methods of coping included providing care and support to other people, caring for personal mental and physical health, believing in solidarity and that COVID-19 was a matter of common concern, and using social media and communication tools to keep social contact with family and friends. Three clusters of coping were generated through factor analysis: (1) social and health factor, with focus on personal health and the social surrounding, (2) activity factor, i.e., being busy with work/studies, finding up-to-date information about COVID-19, while thinking about what one could do rather than what one could not do, and (3) public health factor, which meant trusting health authorities while avoiding misinformation from sources such as social media. The results further show that 56% of the respondents were very, or somewhat satisfied with working from home. Higher levels of satisfaction were observed among respondents aged 35 and above, university employees, and those with a higher level of education.

Our findings show that coping methods from the *health and social factor*, e.g., social interaction, feeling of solidarity and kindness and support to others, were among the most used methods to deal with the challenges of working from home. The COVID-19 pandemic and related restrictions were associated with loneliness and social isolation [41], yet social interaction has a positive effect on wellbeing. The academics adopted several socially oriented coping methods to compensate for the lost face-to-face in-person social contact. Research shows that perceived social support, more so from family, can protect against anxiety [20,42,43,44]. This could explain why efforts to keep in touch with family and friends were prominent among the academics’ methods of coping. This is consistent with finding from other studies among students and university staff [18,45,46]. Most previous studies focus on students; thus, our findings help to illuminate the situation among university staff. However, keeping in touch with friends/family or work colleagues may not be highly prominent in individualistic countries, as shown by results from the Swedish academic setting [36].

Our findings further show that the academics extended their coping beyond mere social interaction, by providing kindness and support to others. This can be explained by research showing that being kind and helpful to others helps to improve the wellbeing of the kind person [47,48,49]. This is irrespective of whether the recipient of the kindness is a stranger or friend/family member [48]. Kindness can be further discussed in relation to altruism. Altruistic behavior, often motivated by empathic feelings, is a selfless act to help and support others often at some cost to oneself, such as time or effort [49]. These types of coping methods are generally used in times of crisis, illness, and loss [50]. Hartman and Morse [51] found that a crisis and trauma can cause solidarity which in turn can motivate empathy-driven altruism.

The COVID-19 pandemic negatively affected people in all walks of life, thus, having solidarity and the belief that “we are all in this together” was important for coping. Jenkins [52] asserts that solidarity, compassion, and empathy experienced via distance tools alleviated the challenges of working from home. Solidarity seemed an important aspect for academics since it was the third most used coping strategy. Solidarity in the context of COVID-19 is explained as “being bound together” and a method for fighting against the viral “enemy” [53]. It is not surprising therefore that altruism was a central part of the academics’ coping methods during the COVID-19 pandemic. Supporting and reaching out to others was a recurring theme throughout the pandemic. Reaching out via social platforms and other distance tools aided individuals to cope with working from home [54,55]. Therefore, while the university staff and students used several methods of coping, the role of social contact, kindness and solidarity cannot be undermined.

Majority of the respondents in our study (56%) were very or somewhat satisfied with working from home. This could be explained by Karasek’s demand-control model that postulates that workers are satisfied with their job when they experience balance between work demands and control over the job [27]. Apart from the obvious benefit of reducing new infections, working from home during the COVID-19 pandemic was associated with positive outcomes such as increased productivity [6,56] and work life balance [56,57]. Ahmadi et al., Arora and Vyas, and Baert et al. [47,58,59] found that employees during the COVID-19 pandemic were overall more satisfied with their jobs. These benefits came with flexibility associated with being able to work undisturbed, saved time due to reduced commuting, and a balance between work and family/private activities. Working from home gives individuals the opportunity to make their own schedule, working in smaller segments, taking breaks when needed and taking care of their household [60]. According to Harpaz [5], working from home can provide autonomy and flexibility. Lopez-Leon et al. [12] discusses that people working from home during the COVID-19 pandemic could combine household activities such as household chores, childcare and home-schooling which could improve the work–life balance. Work–life balance was an important factor for promoting wellbeing and consequent satisfaction with working from home during the COVID-19 pandemic [57].

However, negative effects of working from home are also noticeable. One study reported that 65% of respondents perceived that working from home had a negative effect on team spirit [61]. Moreover, working from home can lead to individuals working more than contracted. This can be due to factors such as increased workload, working more in the evening or weekends, changes in workplace routines and working instead of spending time traveling to and from work [62,63]. Working from home can cause strain within the family due to increased work-family conflicts [64]. Thus, it is not surprising that up to 20% of our respondents were not happy with working from home. In our study, 34% of our respondents reported that they worked more than contracted when working from home during the COVID-19 pandemic. Respondents who worked more than contracted were slightly less satisfied with working from home as compared to those who did not work more than contracted. Working more than contracted seemed therefore to have a negative effect on job satisfaction. This finding is in line with previous research. However, the observed difference in job satisfaction between the two groups was very small. Nevertheless, the finding seems contradictory because 50% of our respondents stated that they often or always kept themselves busy with work because it made them feel good. Put together, our findings seem to indicate that working more than contracted can have both a negative and positive effect on job satisfaction. Therefore, it is necessary to further examine the conditions under which working more than contracted affects job satisfaction. It could be that employees who voluntarily and actively choose to work more than contracted feel good about work as compared to those who feel forced to work more than contracted. Unfortunately, our data do not address the issue of voluntary and forced “working more than contracted”.

The positives and negatives of working from home must not be generalized for all academics; there are differences depending on the characteristics and job demands on different sub-groups. Our findings show that respondents who were older than 35 years, and those with a higher education were more satisfied than the younger ones, students, and those with a lower education level. This may be due to the fact that early and mid-life career researchers, more than full professors and more experienced researchers experienced increased workload and stress during the COVID-19 pandemic [56]. Working from home may provide the opportunity to balance work and family life. However, the possibility that younger researchers have young families may increase the demands on balancing work and family/private life, especially with a shared work-family space. This may especially be the case for students, who often are younger, and must juggle between work, family, and studies. The additional stress of working from home reduces the extent to which they are satisfied with their situation.

## 5. Conclusions

This paper provides novel insight into coping and satisfaction with working from home among a multinational sample of academics during the COVID-19 pandemic. Whereas many studies have investigated coping and job satisfaction generally, this study’s focus on academics and highlight issues that are specific for academics as an occupational group, despite the multicultural sample. Differences in methods of coping and satisfaction with working from home highlight the need for employers to prepare for working from home beyond the COVID-19 pandemic. Despite hesitation about working from home at the beginning of the COVID-19 pandemic, there is positivity about continuing to work from home. Many academics would want to continue working from home after the COVID-19 pandemic.

However, this paper has limitations, mainly due to the sample selection and correlational nature of the study. It is not possible to state with certainty that the observed differences in satisfaction with working from home are due to COVID-19 or specific coping methods. The pandemic has affected all people, albeit in different ways depending on available coping resources, leaving an unexplainable trail. Additionally, the sample is not evenly distributed; for example, men, families without under-aged children, and people with lower education level are under-represented in the sample. This makes it difficult to generalize the results of the study. Furthermore, we studied several counties, yet we were not able to provide analyses specific to the countries we investigated, partly due to the non-symmetrical representation of the countries in the study. Neither did we analyze the different coping methods based on the different sociodemographic variables.

This paper did not set out to investigate differences based on country and culture. However, further studies must consider contextual differences in different academic settings. Furthermore, pandemics and other potentially traumatic events may leave long lasting effects on individuals and communities. Therefore, a longitudinal study design is necessary to investigate the long-term effects of COVID-19, coping, and job satisfaction.

## Figures and Tables

**Table 1:** Sample characteristics (*n* = 674).

Variable	Variable Value	Percentage
*Gender*	Man	34%
	Woman	66%
*Age group*	Younger than 35 years	47%
	Between 35 and 49 years	31%
	50 years or older	22%
*Education level*	University or equivalent	96%
	High school or equivalent	4%
*Occupation*	Employed	64%
	Student	36%
*Civil status*	Married	46%
	Divorced	3%
	Engaged	6%
	Single	35%
	Other	9%
*Children in family*	Yes	64%
	No	36%
*Area of residence*	Capital city	22%
	Medium-large city (not capital)	51%
	Small town, close to large city	19%
	Small town, far from large city	8%

**Table 2:** Factor analysis of methods of coping with challenges of working from home.

	Coping Method	Social and Health Factor	Activity Factor	Public Health Trust Factor
**Social and Health Factor**	I try to provide kindness and support to people around me	0.719	0.154	0.086
	I care for my mental and physical health	0.678	0.117	0.116
	I make sure to have access to medical resources and health services if I need to seek health care	0.652	0.002	0.306
	I have social contact with my family and friends through different social media	0.594	0.231	−0.279
	I believe we are in this together, and with solidarity we can find the best solutions for handling COVID-19	0.5	0.18	0.436
	I have social contact with my colleagues/classmates through different social media	0.458	0.365	−0.452
**Activity Factor**	I give myself a news time limit for each day	−0.019	0.735	0.15
	I make myself busy with my working day because it makes me feel useful	0.213	0.693	−0.104
	I think about what I can do, rather than what I cannot do	0.393	0.473	0.055
	I read/collect information from public health authorities in my country or World Health Organization, and keep myself updated with public health news	0.176	0.525	0.412
**Public Health Trust Factor**	I trust state or local health authorities in my country	0.148	0.065	0.727
	I avoid recommendations that are not from public health authorities in my country, as well as misinformation in social media	0.038	0.067	0.546

## Data Availability

The data supporting the reported results are available at the Faculty of Health and Occupational Studies, University of Gävle. The data are not publicly available to protect informants’ personal information and secrecy.
